# Perforated and bleeding peptic ulcer: WSES guidelines

**DOI:** 10.1186/s13017-019-0283-9

**Published:** 2020-01-07

**Authors:** Antonio Tarasconi, Federico Coccolini, Walter L. Biffl, Matteo Tomasoni, Luca Ansaloni, Edoardo Picetti, Sarah Molfino, Vishal Shelat, Stefania Cimbanassi, Dieter G. Weber, Fikri M. Abu-Zidan, Fabio C. Campanile, Salomone Di Saverio, Gian Luca Baiocchi, Claudio Casella, Michael D. Kelly, Andrew W. Kirkpatrick, Ari Leppaniemi, Ernest E. Moore, Andrew Peitzman, Gustavo Pereira Fraga, Marco Ceresoli, Ronald V. Maier, Imtaz Wani, Vittoria Pattonieri, Gennaro Perrone, George Velmahos, Michael Sugrue, Massimo Sartelli, Yoram Kluger, Fausto Catena

**Affiliations:** 1 Emergency Surgery Department, Parma University Hospital, Parma, Italy; 2 General, Emergency and Trauma Surgery Department, Pisa University Hospital, Pisa, Italy; 3 Scripps Memorial Hospital La Jolla, La Jolla, CA USA; 4 General, Emergency and Trauma Surgery Department, Bufalini hospital, Cesena, Italy; 5 Department of Anesthesia and Intensive Care, Parma University Hospital, Parma, Italy; 6 Department of Clinical and Experimental Sciences, University of Brescia, Brescia, Italy; 7 Tan Tock Seng Hospital, Singapore, Singapore; 8 General Surgery and Trauma Team, ASST Niguarda Milano, University of Milano, Milan, Italy; 9 Royal Perth Hospital, Perth, Australia & The University of Western Australia, Crawley, Australia; 10 Department of Surgery, College of Medicine and Health Sciences, UAE University, Al-Ain, United Arab Emirates; 11 Division of Surgery, ASL VT - Ospedale “Andosilla”, Civita Castellana, Italy; 12 Cambridge Colorectal Unit, Addenbrooke’s Hospital, Cambridge University Hospitals NHS Foundation Trust, Cambridge, UK; 13 Department of Molecular and Translational Medicine, Surgical Clinic, University of Brescia, Brescia, Italy; 14 Department of General Surgery, Albury Hospital, Albury, Australia; 15 General, Acute Care, Abdominal Wall Reconstruction, and Trauma Surgery, Foothills Medical Centre, Calgary, Alberta Canada; 16 Helsinki University Hospital, Helsinki, Finland; 17 Ernest E Moore Shock Trauma Center at Denver Health, Denver, CO USA; 18 University of Pittsburgh, School of Medicine, UPMC – Presbyterian, Pittsburgh, PA USA; 19 Division of Trauma Surgery, School of Medical Sciences, University of Campinas, Campinas, SP Brazil; 20 Department of General and Emergency Surgery, School of Medicine and Surgery, University of Milano-Bicocca, Monza, Italy; 21 Department of Surgery, Harborview Medical Centre, University of Washington, Seattle, USA; 22 Department of Surgery, Sheri-Kashmir Institute of Medical Sciences, Srinagar, India; 23 Trauma, Emergency Surgery and Surgical Critical Care, Massachusetts General Hospital, Boston, USA; 24 Letterkenny University Hospital, Donegal Clinical Research Academy Centre for Personalized Medicine, Donegal, Ireland; 25 Department of Surgery, Macerata Hospital, Macerata, Italy; 26 Department of General Surgery, Rambam Health Care Campus, Haifa, Israel

**Keywords:** Peptic ulcer, High-risk patients, Diagnosis, Non-operative management, Surgery, Antibiotics, Peritonitis, Pancreatitis, Intra-abdominal infection, Technique, Timing, Angiography, Embolization, Guidelines

## Abstract

**Background:**

Peptic ulcer disease is common with a lifetime prevalence in the general population of 5–10% and an incidence of 0.1–0.3% per year. Despite a sharp reduction in incidence and rates of hospital admission and mortality over the past 30 years, complications are still encountered in 10–20% of these patients. Peptic ulcer disease remains a significant healthcare problem, which can consume considerable financial resources. Management may involve various subspecialties including surgeons, gastroenterologists, and radiologists. Successful management of patients with complicated peptic ulcer (CPU) involves prompt recognition, resuscitation when required, appropriate antibiotic therapy, and timely surgical/radiological treatment.

**Methods:**

The present guidelines have been developed according to the GRADE methodology. To create these guidelines, a panel of experts was designed and charged by the board of the WSES to perform a systematic review of the available literature and to provide evidence-based statements with immediate practical application. All the statements were presented and discussed during the 5th WSES Congress, and for each statement, a consensus among the WSES panel of experts was reached.

**Conclusions:**

The population considered in these guidelines is adult patients with suspected complicated peptic ulcer disease. These guidelines present evidence-based international consensus statements on the management of complicated peptic ulcer from a collaboration of a panel of experts and are intended to improve the knowledge and the awareness of physicians around the world on this specific topic. We divided our work into the two main topics, bleeding and perforated peptic ulcer, and structured it into six main topics that cover the entire management process of patients with complicated peptic ulcer, from diagnosis at ED arrival to post-discharge antimicrobial therapy, to provide an up-to-date, easy-to-use tool that can help physicians and surgeons during the decision-making process.

## Introduction

Peptic ulcer disease is common with a lifetime prevalence in the general population of 5-10% and an incidence of 0.1–0.3% per year [1]. Peptic ulceration occurs due to acid peptic damage to the gastro-duodenal mucosa, resulting in mucosal erosion that exposes the underlying tissues to the digestive action of gastro-duodenal secretions. This pathology was traditionally related to a hypersecretory acid environment, dietary factors and stress. However, the increasing incidence of the *Helicobacter pylori* infection, the extensive use of NSAIDs, and the increase in alcohol and smoking abuse have changed the epidemiology of this disease. Despite a sharp reduction in incidence and rates of hospital admission and mortality over the past 30 years [2–8], complications are still encountered in 10–20% of these patients [9, 10]. Complications of peptic ulcer disease include perforation and bleeding and improvement in medical management has made obstruction from chronic fibrotic disease a rare event. A recent review on the epidemiology of complicated peptic ulcer disease [10] found that hemorrhage was by far the most common complication of peptic disease, with a reported annual incidence of hemorrhage in the general population ranging from 0.02 to 0.06%, with sample size-weighted average 30-day mortality of 8.6%. Reported annual incidence of perforation ranges from 0.004 to 0.014%, with sample size-weighted average 30-day mortality of 23.5%. Although perforation is less common, with a perforation:bleeding ratio of approximately 1:6, it is the most common indication for emergency operation and causes about 40% of all ulcer-related deaths [11].

Peptic ulcer disease remains a significant healthcare problem, which can consume considerable financial resources. Management may involve various subspecialties including surgeons, gastroenterologists, and radiologists. Successful management of patients with complicated peptic ulcer (CPU) involves prompt recognition, resuscitation when required, appropriate antibiotic therapy and timely surgical/radiological treatment.

### Notes on the use of the guidelines: aims, targets, and limitations

The Guidelines are aimed to present the state-of-the-art regarding diagnosis and therapeutic options for an optimal management of complicated peptic ulcer. These guidelines are thus intended to improve the knowledge and the awareness of physicians around the world on the specific topic of complicated peptic ulcer, providing an up-to-date tool that can help during the decision-making process. For this reason, the Guidelines are evidence-based and the grade of recommendation is provided to summarize the evidences present in literature. The population considered in these guidelines is adult patients with suspected complicated peptic ulcer disease. The practice Guidelines promulgated in this work do not represent a standard of practice. They are suggested plans of care, based on best available evidence and the consensus of experts but they do not exclude other approaches as being within the standard of practice. For example, they should not be used to compel adherence to a given method of medical management, which method should be finally determined after taking account of the conditions at the relevant medical institution (staff levels, experience, equipment, etc.) and the characteristics of the individual patient. However, responsibility for the results of treatment rests with those who are directly engaged therein, and not with the consensus group.

## Methods

These consensus guidelines are an update of the 2013 WSES position paper on this topic. To create these guidelines, a panel of experts was designed and charged by the board of the WSES to develop questions on six main topics that thoroughly cover the field of this pathology (diagnosis, resuscitation, nonoperative management, surgery, angiography-angioembolization, antimicrobial therapy). Then, leading specialists in the field were asked to perform a thorough search on each of these topics in different databanks (MEDLINE, SCOPUS, EMBASE) for relevant papers between 1985 and June 2018 and a systematic review of the available literature. They were asked to focus their search in order to provide evidence-based answers to every question with immediate practical application and to summarize them in statements. All the statements were presented and discussed during the 5^th^ WSES Congress held in Bertinoro, Italy in June 28th, 2018. For each statement, a consensus among the WSES panel of experts was reached. All the members contributed to the development of the manuscript; the manuscript was reviewed and approved by all the authors.

The present guidelines have been developed according to the GRADE methodology [12, 13].

### Topics and questions

For clarity, we report the six topics together with the questions dividend into each of them.

#### Diagnosis

1. In patients with a suspected perforated  peptic ulcer, which are the appropriate biochemical and imaging investigations that should be requested?
2. In patients with perforated peptic ulcer, what is the clinical value of risk scores such as Boey Score and Pulp score?
3. In patients with suspected bleeding peptic ulcer, which biochemical and imaging investigations should be requested?
4. In patients with suspected bleeding peptic ulcer, what is the diagnostic role of endoscopy?
5. In patients with bleeding peptic ulcer, are the endoscopic findings useful to determine the risk for rebleeding and how do they affect the clinical management?

#### Resuscitation

1. In patients with perforated peptic ulcer, which parameters should be evaluated ad ED referral?
2. In patients with perforated peptic ulcer, which are the appropriate targets for resuscitation (hemoglobin level, blood pressure/heart rate, lactates level, others)?
3. In patients with bleeding peptic ulcer, which parameters should be evaluated at ED referral and which criteria should be adopted to define an unstable patient?
4. In patients with bleeding peptic ulcer, which are the appropriate targets for resuscitation (hemoglobin level, blood pressure/heart rate, lactates level, others)?

#### Non-operative management—endoscopic treatment

1. In patients with perforated peptic ulcer, which are the indications for non-operative management?
2. In patients with perforated peptic ulcer, is there a role for endoscopic treatment?
3. In patients with bleeding peptic ulcer, which are the indications for non-operative management?
4. In patients with bleeding peptic ulcer, which are the indications for endoscopic treatment?
5. In patients with bleeding peptic ulcer, what is the appropriate pharmacological regimen (Erythromycin, PPI, terlipressin, others)?
6. In patients with recurrent bleeding from peptic ulcer, what is the role of non-operative management?

#### Angiography–embolization

1. In patients with bleeding peptic ulcer, which are the indications for angiography?
2. In patients with bleeding peptic ulcer, which are the indications for angioembolization?
3. Should embolization be considered for unstable patients with bleeding peptic ulcer?
4. In patients with recurrent bleeding peptic ulcer, which are the indications for angioembolization?
5. In patients who underwent angioembolization, which are the most appropriate embolization techniques and materials?
6. In patients with bleeding peptic ulcer and non-evident bleeding during angiography is there a role for prophylactic embolization?

#### Surgery

1. In patients with perforated peptic ulcer, which are the indications for surgical treatment and what is the appropriate timing for surgery?
2. In patients with perforated peptic ulcer what is the most appropriate surgical approach (open vs laparoscopy)?
3. In patients with perforated peptic is there a role for sutureless repair?
4. In patients with perforated peptic ulcer and small perforation (< 2 cm), which surgical procedure should be adopted?
5. In patients with perforated peptic ulcer and large perforation (≥ 2 cm), which surgical procedure should be adopted?
6. In patients with perforated peptic ulcer, what is the role of damage control surgery?
7. In patients with bleeding peptic ulcer, which are the indications for surgical treatment and which is the appropriate timing for surgery?
8. In patients with bleeding peptic ulcer, what is the most appropriate surgical approach (open vs laparoscopy) and what are the most appropriate surgical procedures?
9. In patients with bleeding peptic ulcer, what is the role of damage control surgery?

#### Antimicrobial therapy

1. Should antibiotic therapy be prescribed and should anti-fungal therapy be administrated empirically in patients with perforated peptic ulcer?
2. In patients with perforated peptic ulcer, which antimicrobial regimen should be used and what is its correct duration?
3. In patients with bleeding peptic ulcer, which are the indications for antimicrobial therapy and for *Helicobacter pylori* testing?
4. In patients with bleeding peptic ulcer and positive tests for *H. pylori* infection, which are the therapeutic options?

## Perforated peptic ulcer

### Diagnosis

**In patients with a suspected perforated peptic ulcer, which are the appropriate biochemical and imaging investigations that should be requested?**

*In patients with suspected gastroduodenal perforation, we recommend routine laboratory studies and arterial blood gas analysis (strong recommendation based on very low-quality evidences, 1D).*

*In patients with acute abdomen from suspected perforated peptic ulcer, we recommend a CT scan imaging (Strong recommendation based on low-quality evidences, 1C).*

*In patients with acute abdomen from suspected perforated peptic ulcer, we recommend to perform chest/abdominal X-ray as the initial routine diagnostic assessment in case a CT scan is not promptly available (Strong recommendation based on low-quality evidences, 1C).*

*In patients with acute abdomen from suspected perforated peptic ulcer, when free air is not seen on imaging and there is ongoing suspicion of perforated peptic ulcer, we suggest performing imaging with the addition of water-soluble contrast either oral or via nasogastric tube (weak recommendation based on very low-quality evidences, 2D).*

The clinical presentation of gastroduodenal perforation is usually sudden onset of abdominal pain. Localized or generalized peritonitis is typical of perforated peptic ulcer, but may be present in only two-thirds of the patients [14–16]. Thus, physical examination findings may be equivocal and peritonitis may be minimal or absent, particularly in patients with contained and / sealed leak. Laboratory tests are non-specific, although leukocytosis, metabolic acidosis and elevated serum amylase are usually associated with perforation [17]. The first diagnostic investigation is the radiograph of the abdomen and chest, to detect the presence of free abdominal air. Erect and left lateral decubitus X-rays have similar diagnostic accuracy, the latter being better tolerated by patients presenting with peritonitis. The presence of this radiological sign is highly variable across various studies present in literature and ranges between 30 and 85% of perforations. This high variability and the finding that a negative X-ray does not rule out a possible perforation led multiple authors to state that, in case of clear signs of peritonitis, an abdominal CT scan should be the first radiological examination to be performed. However, in the setting of a peripheral hospital without prompt access to a CT scan, the plain X-ray still has a diagnostic role and free air on X-ray associated with a clear history and signs of peritonitis on physical examination is sufficient to justify surgical exploration [9, 14, 15, 18]. An adjunct to plain X-ray could be the administration through a nasogastric tube (NGT) of water-soluble contrast that can detect the presence of a gastro-duodenal perforation. “Point-of-care” ultrasound could also detect free intra-peritoneal, when performed by a trained operator, with the demonstration of air under the abdominal fascia; anyway, its role in the diagnostic work-up of suspected perforated peptic ulcer still needs to be defined. Suspicious CT scan findings include unexplained intraperitoneal fluid, pneumoperitoneum, bowel wall thickening, mesenteric fat streaking, and presence of extraluminal water-soluble contrast. Indeed, CT scan is increasingly taking the main role in diagnosis of perforation, due to the greater sensitivity in detecting free air and to its ability to characterize the site and size of perforation and to exclude other possible causes [15, 18, 19]. However, up to 12% of patients with perforations may have a normal CT scan; in this scenario, the administration of oral water-soluble contrast or via nasogastric tube and performing triple contrast CT scan may improve diagnostic sensitivity and specificity [17].

**In patients with perforated peptic ulcer, what is the clinical value of risk scores such as Boey Score and Pulp score?**

*In patients with perforated peptic ulcer, we suggest to adopt scoring systems (including the Boey, PULP and ASA score) for risk-stratification of patients and to predict outcomes (weak recommendation, based on low-quality evidences, 2C).*

Numerous scoring systems have been designed and validated with the aim of predicting mortality and morbidity in patients with perforated peptic ulcer [20–22]. The Boey score is the most used, followed by the ASA score and the PULP. Boey's score showed an elevated variability in accuracy across the different studies where it was tested. On the other hand, the PULP score is difficult to apply and has not yet been validated outside the initial center. The new PULP score and the ASA score predicted mortality equally well and better than the Boey score, but hypoalbuminemia still remains the strongest single predictor of mortality [20–22].

### Resuscitation

**In patients with perforated peptic ulcer, which parameters should be evaluated ad ED referral?**

*We recommend prompt evaluation and early recognition of the patient with perforated peptic ulcer associated sepsis to prevent further organ failure and to reduce mortality (strong recommendation based on moderate-quality evidences, 1B).*

*We suggest adopting scoring systems (SOFA, qSOFA) to evaluate and assess the severity of the disease in patients with perforated peptic ulcer (Weak recommendation based on low-quality evidences, 2 C).*

Perforated peptic ulcer, with associated peritonitis and sepsis/septic shock, is a medical/surgical emergency requiring rapid evaluation and management [23]. It is crucial to identify parameters to assess the severity of the disease (i.e., to define if a patient is stable or unstable). The latest definition of sepsis/septic shock and related debates/controversies are beyond the scope of this manuscript but are covered in recent papers [24, 25]. The timely recognition of sepsis (i.e., before the occurrence of organ dysfunction) is a priority [25, 26]. During the ED evaluation of every septic patient, several elements should be considered to assess the clinical picture. Specifically, several symptoms (i.e., altered mental state, dyspnea), signs (i.e., tachycardia, tachypnea, reduced pulse pressure, decreased urine output) and laboratory findings (hyperlactatemia, arterial hypoxemia, increased creatinine, coagulation abnormalities) must be evaluated. It is important to keep in mind that these findings may be modified by preexisting disease or medications [27]; for this reason, the collection of clinical history needs to be performed carefully.

Scoring systems, i.e., the sequential organ failure assessment (SOFA) [28] or the quick SOFA (qSOFA) [29], with associated limitations [25, 30–33], are available to assess the severity of the disease.

**In patients with perforated peptic ulcer, which are the appropriate targets for resuscitation (hemoglobin level, blood pressure/heart rate, lactates level, others)?**

*In unstable patients with perforated peptic ulcer, we recommend performing rapid resuscitation to reduce mortality (strong recommendation based on low quality evidences, 1C).*

*In unstable patients with perforated peptic ulcer, we recommend restoring physiological parameters with a mean arterial pressure ≥ 65 mmHg, a urine output ≥ 0.5 ml/kg/h, and a lactate normalization) (strong recommendation based on low-quality evidences,1C).*

*We suggest utlizing different types of hemodynamic monitoring (invasive or not) to optimize fluids/vasopressor therapy and to individualize the resuscitation strategy (strong recommendation based on low quality evidences, 1C).*

Unstable septic perforated peptic ulcer patients need appropriate and rapid (ideally within 1 h) resuscitation to reduce mortality [27, 29]; this must take place simultaneously with surgical consultation, microbiological cultures (blood and other), and antibiotic administration [24, 34]. Primarily, as in any emergency situation, a rapid ABC (airway, breathing, and circulation) evaluation should be done. Secondarily, appropriate targets for resuscitation (the same used for sepsis and septic shock [27, 35]) need to be considered. In general, the most important are:

- Mean arterial pressure (MAP) ≥ 65 mmHg
- Urine output ≥ 0.5 ml/kg/h
- Lactate normalization

Several forms of hemodynamic monitoring (invasive or not) are available to optimize resuscitation and fluid/vasopressors administration. For a more comprehensive approach to sepsis and septic shock, we suggest referring to the last published guidelines of the “Surviving Sepsis Campaign” [35].

### Non-operative management—endoscopic treatment

**In patients with perforated peptic ulcer, which are the indications for non-operative management?**

*In patients with perforated peptic ulcer we suggest against a routinely use of non-operative management; non-operative management (NOM) could be considered in extremely selected cases where perforation has sealed as confirmed on water-soluble contrast study (weak recommendation based on low-quality evidences, 2C).*

Non-operative management (NOM) of perforated peptic ulcer is attractive as it avoids surgery and its resultant morbidity, e.g., wound-related morbidity, postoperative adhesions, etc. The rationale of NOM is that, in the case of small perforations, the ulcer seals by omental adhesions and can then heal and the peritonitis does not need operation [36]. In 1989 Croft et al. conducted a prospective randomized trial [37] comparing emergency surgery and NOM in patients with a clinical diagnosis of perforated peptic ulcer: 83 patients were entered in the study over a period of 13 months and were randomly assigned to one the two study groups. In the NOM group, 11 patients (28 percent) had no clinical Improvement after 12 h and required an operation. The overall mortality rates in the two groups were similar (two deaths in each, 5%), and did not differ significantly in the morbidity rates (40% in the surgical group and 50% in the nonsurgical group). The hospital stay was 35% longer in the group treated conservatively and patients over 70 years old were less likely to respond to conservative treatment than younger patients (*p* < 0.05). Songne et al. in 2004 [38] conducted a prospective trial of 82 consecutive patients with diagnosis of perforated peptic ulcer; they initially underwent NOM and clinical improvement was achieved in 54% of patients after NOM. In multivariate analysis, the factors independently related to NOM failure were size of pneumoperitoneum, heart rate > 94 bpm, and abdominal meteorism (defined as distended bowel loops). In conclusion, the most important factors regarding the feasibility of NOM for perforated peptic ulcer are normal vital signs in a stable patient and whether the ulcer itself has sealed as confirmed by a water-soluble contrast study: if there is a free leak of contrast, surgery is needed. On the other hand, NOM could be considered if no contrast extravasation is present and the patient does not have signs of peritonitis or sepsis.

The essential pre-requisites and components of non-operative management of PPU can be grouped as “R”s [39]:

- Radiologically undetected leak
- Repeated clinical examination
- Repeated blood investigations
- Respiratory and renal support
- Resources for monitoring and
- Readiness to operate

NOM includes: nil by mouth; intravenous hydration; decompression via nasogastric tube; anti-secretory and PPI therapy; intravenous antibiotics; and follow-up endoscopy at 4–6 weeks. Mortality increases with every hour of delay to surgery, and hence, NOM must be carefully selected. Surapaneni et al. have shown nil mortality in patients who were operated within 24 h of onset of symptoms as compared to surgery beyond 48 h of onset of symptoms [40]. Buck et al. in 2688 Danish patients have shown that every hour of delay from admission to surgery was associated with an adjusted 2.4% decreased probability of survival compared with the previous hour [41]. Elderly patients may experience paradoxical higher mortality if non-operative management fails and caution is advised in patients > 70 years of age.

**In patients with perforated peptic ulcer is there a role for endoscopic treatment?**

*In patients with perforated peptic ulcer, we suggest to avoid endoscopic treatment such clipping, fibrin glue sealing, or stenting (Weak recommendation based on low-quality evidences, 2C)*

Closure of acute iatrogenic perforations with endoscopic clips is described [42, 43]; however, clips may not be effective in perforated ulcer cases due to fibrotic tissue with loss of compliance. Combined laparoscopic-endoscopic approaches for perforated ulcer closures have been described [44, 45]. Bergstrom et al. [46] present a case series of eight patients with perforated duodenal ulcers treated with covered self-expandable metal stents and the results indicate that, in very selected patients or in cases where surgical closure will be difficult, gastroscopy with stent placement could be performed during laparoscopy, followed by laparoscopic drain placement. In patients with severe co-morbidity or delayed diagnosis, gastroscopy and stent placement followed by radiologically guided drain placement could be an alternative to more standard treatment. Endoscopic snaring of omentum and pulling is also described as an effective adjunct along with duodenal plication. Furthermore, endoscopy also allows performing a biopsy and rule out gastric outlet obstruction in case of large perforations. In spite of these case series, all the above reported modalities are not recognized as standard approaches to perforated peptic ulcer and need further validation.

### Surgery

**In patients with perforated peptic ulcer, which are the indications for surgical treatment and what is the appropriate timing for surgery?**

*In patients with perforated peptic ulcer with significant pneumoperitoneum or extraluminal contrast extravasation or signs of peritonitis, we recommend operative treatment (Strong recommendation based on low-quality evidences, 1C)*

*We recommend performing surgery as soon as possible, especially in patients with delayed presentation and patients older than 70   years old (strong recommendation based on moderate-quality evidences, 1B)*

The feasibility of NOM should be weighed with the evidence that an increase in surgical delay significantly impairs surgical outcome. In fact, a cohort study performed in 2013 from the Danish Clinical Register of Emergency Surgery [41] showed that, over the first 24 h after admission, each hour of surgical delay beyond hospital admission was associated with an adjusted 2.4% decreased probability of survival compared with the previous hour, over the entire observation period. Other studies highlighted the importance of a prompt surgical approach to PPU: a retrospective single-center study by Lunevicious et al. [47] showed an increase in the suture leakage rate after a delay in presentation > 9 h, while a recent prospective single-center study on 101 patients with peritonitis from peptic ulcer perforation who underwent laparotomy and simple closure with omental patch found that a perforation-to-surgery interval longer than 36 h was significantly associated with an increase in postoperative mortality [48]. Furthermore, a systematic review [49] performed in 2010 including fifty studies with 37 prognostic factors comprising a total of 29,782 patients provided strong evidence for an association of older age, comorbidity, and use of NSAIDs or steroids with mortality; shock upon admission, preoperative metabolic acidosis, tachycardia, acute renal failure, low serum albumin level, high ASA score, and preoperative delay > 24 h were also associated with poor prognosis. Limiting pre-operative delay thus seems to be of great importance.

**In patients with perforated peptic ulcer, which is the most appropriate surgical approach (open vs laparoscopy)?**

*In stable patients with perforated peptic ulcer, we suggest a laparoscopic approach. An open approach is recommended in the absence of appropriate laparoscopic skills and equipment (weak recommendation based on moderate-quality evidences, 2B).*

*In unstable patients with perforated peptic ulcer, we recommend open surgery (strong recommendation based on very low-quality of evidences, 1D)*

A recent meta-analysis from Cirocchi et al. [50] compared laparoscopic to open surgery for patients with perforated peptic ulcer: their search identified 8 RCTs for a total of 615 patients (307 patients undergoing laparoscopic repair and 308 patients undergoing open repair); however all the included studies were at high risk of bias. The comparison reported a significant advantage of laparoscopic repair with less postoperative pain in the first 24 h after surgery and less postoperative wound infections. No significant differences between laparoscopic and open surgery were found for overall postoperative mortality, leak of the suture repair, intra-abdominal abscesses and reoperation rate. This is the strongest evidence present so far the literature and suggests it is reasonable to pursue a laparoscopic approach for stable patients and in the presence of appropriate surgical skills.

The effects of increased intra-abdominal pressure and hypercarbia due to CO2 insufflation during laparoscopy are well known (increased systemic vascular resistance, mean arterial pressure, afterload, heart rate, caval pressures, respiratory rate, peak airways pressure, PaCO2; reduced stroke volume, venous return, cardiac output, thoracic compliance, pH) [51] and preclude a laparoscopic approach to hemodynamically unstable patients or patients with severe cardiovascular or pulmonary comorbidity.

**Is there a role for sutureless repair in patients with perforated peptic ulcer?**

*Based on the available literature, no recommendation could be made about the sutureless repair.*

Sutureless repair was proposed with the rationale to shorten operative time and to simplify the surgical technique, making it easily performed by those who have limited experience with laparoscopic surgery. However, it has not gained a wide acceptance due to its high leakage rate compared to suture repair. A prospective study conducted from January 1992 to December 1998 included 374 patients with perforated peptic ulcer [52]; 219 patients were treated by open suture repair, 109 patients received laparoscopic fibrin glue repair and the remaining 46 patients were treated by laparoscopic suture repair. Laparoscopic fibrin glue repair was initially attempted in 149 patients but 40 required conversion to suture repair. The overall conversion rates for laparoscopic fibrin glue repair and laparoscopic suture repair were 27 and 15%, respectively. The main reasons for conversion were a large (1 cm or more) ulcer perforation and failure to locate the perforation site. The overall leak rates after laparoscopic glue repair and laparoscopic suture repair were 16 and 6% respectively and the reoperation rates for clinical leaks after laparoscopic glue repair and laparoscopic suture repair were 10 and 4% respectively. On the other hand, a retrospective cohort study performed from January 2008 to December 2012 found conflicting results [53]: 107 patients were included, 64 underwent laparoscopic repair with a sutureless on-lay omental patch, and 43 were treated by laparoscopic sutured omental patch. High-risk patients with Boey scores of 2 and 3 or those with perforations larger than 10 mm were excluded. The time to water intake was significantly shorter for patients who had repair with a sutureless omental patch (*p* = 0.007), as well as the mean hospital stay (*p* = 0.007). All patients in both groups survived to the end of the study and no patient experienced leakage after the operation. The evidences listed above are based on low quality studies and do not allow us to make a recommendation for its routine application.

**In patients with perforated peptic ulcer and small perforation (< 2 cm), which surgical procedure should be adopted?**

*In patients with perforated peptic ulcer smaller than 2 cm, we suggest performing primary repair. No recommendation can be made whether the use of an omental patch can provide further protection of the repair (weak recommendation based on low-quality evidences, 2C)*

Historically, repair with the adjunct of an omental patch was considered the “standard” laparoscopic procedure for perforated peptic ulcer repair. This belief is a now matter of debate as multiple studies showed the addition of an omental patch does  not add benefits to a simple suture repair, but it significantly increases the operation time.

Multiple retrospective single-center studies support these findings. Lin et al. [54] analyzed 118 patients with PPU who underwent laparoscopic repair with simple closure (*n* = 27) or omentopexy (*n* = 91) and found Three closure leakage: 1 after simple closure and 2 after omentopexy, but no patient died. After matching, the simple closure and omentopexy groups had comparable results regarding leakage rate. Comparison of the operating time in the 4.0- and 5.0–12-mm groups reported that the simple closure took less time than omentopexy for perforations smaller than 12 mm. Abd Ellatif and colleagues [55] enrolled 179 consecutive patients with PPU who were treated by laparoscopic repair; 108 patients with the omental patch technique and 71 with laparoscopic simple repair. Operative time was significantly shorter in the non-patch group and no patient was converted to laparotomy. There was no difference in age, gender, ASA score, surgical risk (Boey’s) score, and incidence of co-morbidities between two groups and both groups was comparable in terms of hospital stay, time to resume oral intake, postoperative complications and surgical outcomes. Lo et al. retrospectively identified 73 patients undergoing PPU laparoscopic repair, 26 received simple closure repair and 47 received simple closure plus omental patch. There was no difference in age, gender, ASA score, Boey risk score, incidence of co-morbidities, Mannheim Peritonitis index, median operation time or length of stay. Again, they stated that, in terms of leakage rate and surgical outcome, the maneuver to cover an omental patch on the repaired PPU did not show additional advantage compared to simple closure alone [56]. A multicenter non-randomized retrospective study [57] further strengthens these findings: between 2009 and 2013, 297 patients with PPU underwent a laparoscopic procedure in eight Romanian surgical centers. Primary suture repair was performed in 145 patients (48.8%), primary suture repair with omentopexy in 146 patients (49.2%) and the remaining 6 patients were converted to open surgery. The univariate complications rate analysis they performed found no significant association (*p* = 0.634; Fisher’s exact test) between the type of the repair and the rate of complications. A prospective non-randomized study by Ates et al. compared laparoscopic simple closure with conventional omental patch open repair for perforated peptic ulcer. Of the 35 patients enrolled, none experienced operative complications nor postoperative leak or residual intra-abdominal abscess [58]. On the other hand, multiple retrospective studies highlight low postoperative leak rates with the omental patch technique, even in case of perforations up to 2 cm in diameter [59]. Multiple authors suggest the adjunct of an omental patch in case of large ulcers with friable edges, to reduce the risk of the suture cutting through the edges of the ulcer [60].

In light of the above, we cannot suggest the routine application of the omental patch because of the longer operative time, the need for advanced laparoscopic skills and the similar results after simple closure, but it could be considered a viable option in selected cases.

**In patients with perforated peptic ulcer and large perforation (≥ 2 cm), which surgical procedure should be adopted?**

*We suggest a tailored approach based upon the location of the ulcer for the treatment of perforated peptic ulcer larger than 2 cm. In case of large gastric ulcers that raise the suspicion of malignancy, we suggest resection with contextual operative frozen pathologic examination whenever possible. In case of large duodenal ulcers, we suggest considering the need of resections or repair plus/minus pyloric exclusion/external bile drainage. We recommend duodenostomy only in extreme circumstances (weak recommendations based on very low-quality evidences, 2D).*

While the treatment of a small ulcer is relatively straightforward, the treatment of giant peptic ulcers (diameter > 2 cm) poses different challenges according to the anatomical location. Furthermore, large gastric ulcers should always raise the suspicion of malignancy [61]. The spontaneous perforation of gastric cancer is a rare complication, occurring in 1% of patients with gastric cancer, and it has been reported that about 10–16% of all gastric perforations are caused by gastric carcinoma [62]. Besides this, there are no specific surgical treatment recommendations since the site of perforation and the secondary effects on the surrounding anatomical structures must direct the necessary interventions. The gastric location is usually easier to treat when compared to the duodenal location and gastric resection and reconstruction should be the surgical choice for the treatment of perforated gastric ulcers larger than 2 cm. On the other hand, only the first portion of the duodenum can be resected easily without risk of injuring the bile duct or the pancreatic head. Antrectomy plus or minus D1–D2 resection with diversion is the classic and most commonly described intervention, if the ampullary region is not involved [63]. The proximity of the defect and its relation to the common bile duct and ampulla of Vater must also be thoroughly investigated and intraoperative cholangiography may even be necessary to verify common bile duct anatomy. Several different procedures, such as a jejunal serosal patch, Roux en-Y duodenojejunostomy, pyloric exclusion, and several variations of omental plugs [64] have been described for large duodenal defects when the defect is felt too large to perform a primary repair. In large ulcers, leak rates up to 12% have been reported from attempted closure with an omental patch procedure [65]. These patients also frequently present in septic shock when the amount of peritoneal spillage is large. This factor alone should significantly influence the choice of operative intervention, because a definitive resectional approach for ulcers involving the ampullary area (i.e., Whipple procedure or similar) is usually not recommended in patients with peritonitis, because of the high physiological impact of these procedures and the great risk of postoperative complications. In these cases, a damage control procedure (such as pyloric exclusion with gastric decompression via a nasogastric tube or a gastrostomy and an external biliary diversion via T-tube) will likely be the safest and most appropriate operation for the patient [66]. Duodenostomy (e.g., over Petzer tube) should be used only as a last resort, in the presence of giant ulcers with severe tissue inflammation and when mobilization of the duodenum is not possible and the patient is in severe septic shock with hemodynamic instability.

**In patients with perforated peptic ulcer, what is the role of damage control surgery?**

*In patients with septic shock from a perforated peptic ulcer and signs of severe physiological derangement, we suggest a damage control strategy (Weak recommendation based on very low-quality of evidences, 2D)*

In severe peritonitis, some patients may experience disease progression to severe sepsis and septic shock experiencing progressive organ dysfunction, hypotension, myocardial depression, and coagulopathy and a staged approach may be required. If the patient is not in a condition to undergo a definitive repair and/or abdominal wall closure, due to mandatory conditions requiring an open abdomen, the intervention should be abbreviated due to suboptimal local conditions for healing and global susceptibility to spiraling organ failure [67]. Such mandatory conditions include physical inability to close the abdominal fascia without tension, a decision to leave intra-abdominal packing, or a decision to leave blind bowel loops to expedite the procedure. Committing a patient to an open abdomen however has significant risks including the most feared enteroatmospheric fistula which has been reported to be more common in emergency general surgery patients than trauma patients. “Source control” of intra-abdominal contamination remains a discretionary reason to leave the abdomen open, recognizing that “inability to achieve source control” is a frequently quoted but poorly objectified concept in emergency general surgery. Although upper gastrointestinal perforations are often less catastrophic than lower gastrointestinal contaminations, when the patient responded with immunological activation and systemic sepsis, they are suffering from severe complicated intra-abdominal sepsis. If these conditions are met, then we suggest participation and potential enrollment in the COOL Trial [68–70] Closed or Open after Laparotomy (COOL) study (https://clinicaltrials.gov/ct2/show/NCT03163095) to help provide better guidance for clinicians in the future treating such challenging patients. In general, anastomoses should be avoided in the presence of hypotension or hemodynamic instability, especially if the patient requires vasopressors. After copious abdominal irrigation, a temporary abdominal closure device can be placed if there are mandatory factors dictating an OA or if the patient is randomized to this therapy in the COOL trial. The patient can then be resuscitated appropriately in the ICU. The surgeon can return to the OR for re-exploration, restoration of continuity and closure of the abdomen once the patient is hemodynamically stable. We refer you to the WSES guidelines on Open Abdomen management for further information [67].

### Antimicrobial therapy

**Should antibiotic therapy be prescribed and should anti-fungal therapy be administrated empirically in patients with perforated peptic ulcer?**

*In patients with perforated peptic ulcer, we recommend the administration broad-spectrum antibiotics (strong recommendation based on low-quality evidences, 1C)*

*We recommend the collection of samples for microbiological analysis for both bacteria and fungi in all patients undergoing surgery with subsequent antibiotic therapy adjustment (strong recommendation based on low-quality evidences, 1C*

*We suggest not to administer antifungal agents as standard empiric therapy in patients with perforated peptic ulcer. Antifungal should be administrated in patients at high risk for fungal infection (e.g., immunocompromised, advanced age, comorbidities, prolonged ICU-stay, unresolved intra-abdominal infections) (weak recommendation based on low-quality evidences, 2C)*

The perforation of a peptic ulcer almost invariably leads to peritonitis due to the spillage of gastroduodenal content into the peritoneal cavity; this event brings a great burden of morbidity, which ranges from 17% to 63%, and is usually represented by pulmonary and wound infections [66]. Bacteria involved in peritoneal sepsis vary according to the etiology of the peritonitis, including the site of perforation. They are usually represented by gram-positive, gram-negative as well as anaerobic species [71]. Samples of peritoneal fluid should be collected in perforated patients because fungal infections after perforation are common and are associated with longer hospital stay, higher rate of surgical site infections (SSI), and increased mortality, as reported in a prospective study by Shan and coworkers [72]. In the same way, Prakash and coworkers [73] demonstrated in a prospective study on 84 patients undergoing surgery for perforation peritonitis that mortality was higher in patients having positive peritoneal fluid cultures (*p* < 0.001) compared with those with negative cultures, and in those subjects having mixed bacterial and fungal positive cultures compared with those with isolated bacterial cultures (*p* < 0.001).

Notwithstanding positive peritoneal fungal culture is a significant risk factor for adverse outcome in patients with PPU [72, 73], the addition of an antifungal therapy to a broad-spectrum antibiotic therapy is still a matter of debate [74]. While antifungal therapy is recommended for hospital-acquired infections and in patients critically ill or severely immunocompromised [75], in case of community-acquired fungal infection, it has been suggested that antifungal therapy should be reserved for only clinically severe cases [76].

In a retrospective analysis of 133 patients admitted to the emergency department for abdominal pain due peptic perforation, Li and coworkers [74] demonstrated that there was not a statistically significant difference in survival rate between patients who received antifungal therapy and those who did not and that, on a multivariate analysis, only shock on admission and an APACHE score higher than 20 were independent risk factors for a poor outcome. According to this evidence, antifungal therapy does not benefit patients suffering from PPU peritonitis with *Candida* spp. isolated from peritoneal fluid cultures in general, and antifungal therapy should be reserved for patients who are critically ill and/or severely immunocompromised.

**In patients with perforated peptic ulcer, which antimicrobial regimen should be used and what is its correct duration?**

*In patients with perforated peptic ulcer, we recommend to start as soon as possible an empiric broad-spectrum antibiotic regimen against a mixture of Gram-negative, Gram-positive, and anaerobic bacteria, possibly after peritoneal fluid has been collected (Strong recommendation based on low-quality evidences, 1C)*

*In patients with perforated peptic ulcer, we suggest a short-course (3–5 days or until inflammatory markers normalize) antibiotic therapy (weak recommendation based on low-quality evidences, 2C).*

Perforated peptic ulcer peritonitis is by definition poly-microbial. Gram-negative and Gram-positive as well as anaerobic bacteria and yeasts can be isolated from peritoneal fluid cultures. Antimicrobial therapy, together with adequate source control, plays a pivotal role in the management of patients with peritonitis, especially in those who are immunocompromised. As stated in a previous published paper [71], an empiric broad-spectrum antimicrobial therapy should be started as soon as possible, and possibly after peritoneal fluid sample collection, irrespective of the presence of severe sepsis or septic shock. In these patients, a de-escalation approach is warranted, to avoid the onset of microbial resistances and to promptly treat eventual sepsis. The empiric antimicrobial regimen should be single or combined, according to the range requirements of antimicrobial coverage and the risk factors for major resistance patterns [76].

Modification of the drug regimen becomes possible when cultures are available, and clinical status can be better assessed. If inflammatory markers do not improve, it is mandatory to rule out other extra-abdominal sources of infections or different pathogens [71]. As widely accepted [71], a beta-lactam/beta-lactamase inhibitor can be used as first-line therapy in case of intra-abdominal infections, due to its vigorous in vitro activity against gram-positive, gram-negative, and anaerobic bacteria [77]. The principles of empiric antibiotic treatment should be defined according to the most frequently isolated bacteria, always taking into consideration the local trend of antibiotic resistance. In this era of prevalent drug-resistant microorganisms, the threat of resistance is a source of major concern that cannot be ignored. In the past 20 years, incidence of healthcare-associated IAIs caused by MDROs has risen dramatically [78], probably in correlation with escalating levels of antibiotic exposure and increasing frequency of patients with one or more predisposing conditions, including elevated severity of illness, advanced age, degree of organ dysfunction, low albumin levels, poor nutritional status, immunosuppression, presence of malignancy, and other comorbidities. The first step in determining potential resistance patterns of a given infection is by establishing whether the infection is community-acquired or healthcare-associated (nosocomial). The spectrum of microorganisms involved in nosocomial infections is significantly broader than in community-acquired infections.

Quinolone resistance, prevalence of ESBL-producing bacteria, prevalence and mechanisms of carbapenem resistance in the local environment, and the place of recent traveling should be always taken into account when an antibiotic therapy is administered empirically. Generally, the most important factors in predicting the presence of resistant pathogens in intra-abdominal infections are acquisition in a healthcare setting (particularly if the patient becomes infected in the ICU or has been hospitalized for more than 1 week), corticosteroid use, organ transplantation, baseline pulmonary or hepatic disease, and previous antimicrobial therapy [78]. In patients with IAIs, when patients are not severely ill and when source control is complete, a short course (3–5 days) of postoperative therapy is suggested. In 2015, a prospective study on appropriate duration of antimicrobial therapy was published [79]: the study randomized 518 patients with IAIs and adequate source control to receive antibiotics until 2 days after the resolution of fever, leukocytosis, and ileus, with a maximum of 10 days of therapy (control group), or to receive a fixed course of antibiotics (experimental group) for 4 ± 1 calendar days. In patients with intra-abdominal infections who had undergone an adequate source control procedure, the outcomes after fixed-duration antibiotic therapy (approximately 4 days) were similar to those after a longer course of antibiotics (approximately 8 days) that extended until after the resolution of physiological abnormalities. In this study, most patients were not severely ill.

If yeast are isolated in the peritoneal fluid culture, the antifungal regimen should be selected according to the clinical and immunological status of the patient, severity of disease, prior exposure to other antifungal therapies, and type of infection (community-acquired vs. hospital-acquired) [80]. The duration of hospital stay is a concern, because prolonged stay is associated with antifungal resistance of *Candida* strains [81]. Moreover, biofilm formation of fungi usually goes along with significant changes in virulence and resistance because, once embedded into biofilm, fungi become more protected against the fungicidal/fungistatic effect of drugs.

Four classes of antifungal drugs are available [82]:

1. Azoles (fluconazole, itraconazole, voroconazole, and posaconazole), with fungistatic action against most *Candida* spp.;
2. Echinocandins (caspofungin, micafungin, anidulafungin), with fungicidal effect;
3. Polyenes (deoxycholate and liposomal formulations of amphotericin B), with fungicidal effect but moderate peritoneal penetration;
4. Flucytosine, only used in combination with another antifungal agent in difficult-to-treat cases, because of the high risk of resistance.

Fungistatic drugs should be used in critically ill patients at low-risk for invasive *Candida* infections, without prior exposure to azoles, and the therapy should be administered for 7-10 days or until definitive negative fluid cultures. In high-risk patients with or without prior exposure to azoles, echinocandins should be preferred. The duration of treatment depends on the extent of organ involvement. If candidemia is detected, the administration should be prolonged at least 14 days after the end of episode [82].

Following we report the suggested antibiotic regimens according to WSES guidelines on intra-abdominal infections.

*Community-acquired*

*1) Empiric antibiotic regimens for non-critically ill patients with IAIs and normal renal function:*

- *Amoxicillin/clavulanate 1.2-2.2 g 6-hourly or ceftriaxone 2 g 24-hourly + metronidazole 500 mg 6-hourly or cefotaxime 2 g 8-hourly + metronidazole 500 mg 6-hourly*
- *In patients with beta-lactam allergy: ciprofloxacin 400 mg 8-hourly + metronidazole 500 mg 6-hourly*
- *Patients at risk for infection with community-acquired ESBL-producing Enterobacteriacea: ertapenem 1 g 24 hourly or tigecycline 100 mg initial dose, then 50 mg 12-hourly*

*2) Empiric antibiotic regimens for critically ill patients with IAIs and Normal renal function:*

- *Piperacillin/tazobactam 4.5 g 6-hourly or cefepime 2 g 8-hourly + metronidazole 500 mg 6-hourly*
- *patients at risk for infection with community-acquired ESBL-producing Enterobacteriacea: meropenem 1 g 8-hourly or doripenem 500 mg 8-hourly or imipenem/cilastatin 1 g 8-hourly*

*3) If antifungal therapy is indicated:*

- *Fluconazole (LD 12 mg/kg BW-800 mg; MD 6 mg/kg/day) should be given in critically ill patients, with community-acquired Candida peritonitis, no prior azole exposure, low-risk for infections with fluconazole-resistant Candida spp., as prophylaxis to prevent invasive infections*
- *Echinocandin antifungals are recommended as first-line therapy for invasive infections, and candidemia in non-neutropenic critically ill patients*
- *Amphotericin B (3–5 mg/day) should be considered if alternative therapy is not available or in case of intolerance to echinocandin or azoles*

*Healthcare-associated*

*1) Empiric antimicrobial regimens for non-critically ill patients with IAIs and normal renal function:*

- *Piperacillin/tazobactam 4.5 g 6-hourly*
- * In patients at higher risk for infection with MDROs including recent antibiotic exposure, patient living in a nursing home or long-stay care with an indwelling catheter or postoperative infections*
*○ Meropenem 1 g 8-hourly +/− ampicillin 2 g 6-hourly or*
*○ Doripenem 500 mg 8-hourly +/− ampicillin 2 g 6-hourly or*
*○ Imipenem/Cilastatin 1 g 8-hourly or*
*○ As a carbapenem-sparing regimen piperacillin/tazobactam 4.5 g 6-hourly + tigecycline 100 mg initial dose, then 50 mg 12-hourly*

*2) Empiric antimicrobial regimens for critically ill patients with IAIs normal renal function*

- *Meropenem 1 g 8-hourly or*
- *Doripenem 500 mg 8-hourly or*
- *Imipenem/cilastatin 1 g 8-hourly*

*+*

- *Vancomycin 25–30 mg/kg loading dose then 15–20 mg/kg/dose 8-hourly or*
- *Teicoplanin 12 mg/kg 12-hourly times 3 loading dose then 12 mg/kg 24-hourly*

*3) In patients at risk for infection with vancomycin-resistant Enterococci (VRE) including patients with previous enterococcal infection or colonization, immunocompromised patients, patients with long ICU stay, or recent vancomycin exposure:*

- *Linezolid 600 mg 12-hourly or*
- *Daptomycin 6 mg/kg 24-hourly*

## Bleeding peptic ulcer

### Diagnosis

**In patients with suspected bleeding peptic ulcer, which biochemical and imaging investigations should be requested?**

*In patients with suspected bleeding peptic ulcer, we recommend blood-typing, determinations of hemoglobin, hematocrit and electrolytes, and coagulation assessment (strong recommendation based on very low-quality evidences, 1D).*

*In patients with suspected bleeding peptic ulcer, when endoscopy is not available, we suggest performing contrast-enhanced CT scan (weak recommendation based on very low-quality evidences, 2D)*

Peptic ulcer is still the primary cause of non-variceal upper gastrointestinal bleeding and hypovolemic shock or its consequences is a major cause of mortality in acute upper gastrointestinal bleeding [1, 83]. In the acute setting, with the suspicion of bleeding peptic ulcer, blood tests that include blood-typing and cross-matching with determinations of hemoglobin, hematocrit, electrolytes, and coagulation assessment should be performed in all patients. Alteration of coagulation with INR greater than 1.5 is associated with an increased risk of mortality [84].

Data are limited in the literature on the use of CT-scan in the evaluation of gastrointestinal bleeding. Given the assumption that gastroscopy is the first diagnostic step, in patients where it is negative or not feasible, CT-scan may be a valuable tool to detect the site and the degree of the bleeding. Otherwise, CT angiography is the first-line investigation of choice for undifferentiated major gastrointestinal hemorrhage (being particularly useful for the localization of small and large intestinal acute hemorrhage). There are increasing data to suggest that CT-scan should be the “next step” investigative procedure in cases of active GI hemorrhage [85, 86].

**In patients with suspected bleeding peptic ulcer, what is the diagnostic role of endoscopy?**

*In patients with suspected bleeding peptic, ulcer, we recommend performing endoscopy as soon as possible, especially in high-risk patients (Strong recommendation based on low-quality evidences, 1C)*

Gastroscopy must take place as soon as possible. Many studies, including a meta-analysis of randomized controlled trials [87], have shown the role of gastroscopy in reducing rebleeding, need for surgery, and mortality. Early endoscopy done within 24 h provides both an effective therapy of the bleeding and prognostic information based on endoscopic stigmata [88, 89].

**In patients with bleeding peptic ulcer, are the endoscopic findings useful to determine the risk for rebleeding and how do they affect the clinical management?**

*We suggest guiding management decisions according to stigmata of recent hemorrhage during endoscopy because they can predict the risk of further bleeding (strong recommendation based on low-quality evidences, 1C)*

The gastroscopy findings can be classified using the modified Forrest classification. With the identification of lesions with high-risk stigmata, it is possible to stratify the risk of rebleeding, the need for intervention, and mortality [89, 90]. Furthermore, gastroscopy is essential in identifying patients with a low risk that may be discharged early [87, 88]. Numerous scores have been tested to predict the need for surgery and gastroscopy, the Glasgow-Blatchford Score (GBS), the Rockall score, and the AIMS65 being the most widely evaluated and adopted. Risk stratification should identify high-risk patients for early intervention and reduce the duration of hospital stay for low-risk patients [91, 92].

### Resuscitation

**In patients with bleeding peptic ulcer, which parameters should be evaluated at ED referral and which criteria should be adopted to define an unstable patient?**

*We recommend a rapid and careful surgical/medical evaluation of bleeding peptic ulcer disease patients to prevent further bleeding and to reduce mortality (strong recommendation based on very low-quality evidences, 1D)*

*We recommend evaluating several elements (symptoms, signs, and laboratory findings) to assess the stability/instability of patients with bleeding peptic ulcer at ED referral (strong recommendation based on low quality evidences, 1C)*

*In patients with bleeding peptic ulcer, we suggest evaluating patients according to Rockall and Glasgow-Blatchford scoring systems to assess the severity of the disease and to guide therapy (weak recommendation based on low-quality evidences, 1C).*

Bleeding peptic ulcer disease is a clinical emergency requiring a rapid surgical/medical evaluation to assess the stability of the clinical picture; the approach is similar to the bleeding trauma patient [93]. In this regard, we suggest referring to the last edition of the European guideline on management of major bleeding and coagulopathy following trauma [94]. The parameters that should be assessed at ER referral are the same as reported in the American College of Surgeons Advanced Trauma Life Support (ATLS) (American College of Surgeons Committee on Trauma. ATLS® Student Manual 10th Edition; 2018) classification of blood loss (heart rate, blood pressure, pulse pressure, respiratory rate, urine output, Glasgow Coma Scale score, and base deficit). Moreover, it is very important to take an accurate medical history [93] especially regarding:

- Drugs and diseases that may affect the coagulation status (i.e., antiplatelets, anticoagulants, hepatic failure)
- Cardiac (i.e., coronary artery disease) and pulmonary diseases that may make patients more susceptible to adverse effects of anemia
- Neurological diseases (i.e., dementia) that may predispose patients to pulmonary aspiration of gastric contents.

Several scoring systems are available for the evaluation of patients with upper gastrointestinal bleeding. The Rockall score [95] can be utilized to identify patients at risk of adverse outcomes where the Glasgow-Blatchford bleeding score [96] identifies patients needing interventions such as blood transfusions or endoscopy.

**In patients with bleeding peptic ulcer, which are the appropriate targets for resuscitation (hemoglobin level, blood pressure/heart rate, lactates level, others)?**

*We recommend several resuscitation targets, similar to those of damage control resuscitation in the bleeding trauma patient (weak recommendation based on low-quality evidences, 1C).*

*In patients with bleeding peptic ulcer, we recommend to maintain an Hb level of at least > 7 g/dl during the resuscitation phase (strong recommendation based on moderate-quality evidences, 1B).*

Early resuscitation of patients with upper gastrointestinal bleeding is of paramount importance to reduce mortality; this must proceed simultaneously with endoscopic and surgical procedures [97]. A rapid ABC (airway, breathing, and circulation) evaluation should be done immediately. Appropriate targets for resuscitation in bleeding peptic ulcer patients can be considered the same used in bleeding trauma patients (systolic blood pressure of 90–100 mmHg until major bleeding has been stopped; normalization of lactate and base deficit; hemoglobin 7–9 g/dl; correction/prevention of coagulopathy); for this reason, we refer to the abovementioned guideline [94]. Regarding hemoglobin level, a randomized controlled trial comparing the efficacy and safety of a restrictive transfusion strategy (transfusion with an Hb > 7 g/dl) with those of a liberal transfusion strategy (transfusion with an Hb > 9 g/dl) in severe acute gastrointestinal bleeding has been performed [98]. The restrictive strategy, compared with the liberal strategy, has been significantly associated with a better outcome.

### Non-operative management—endoscopic treatment

**In patients with bleeding peptic ulcer, which are the indications for non-operative management?**

*In patients with bleeding peptic ulcer, we recommend non-operative management as the first line of management after endoscopy (strong recommendation based on low-quality evidences, 1C).*

Non-operative management of bleeding peptic ulcer incorporates principles of ABCDE [99]:

- Airway control
- Breathing—ventilation and oxygenation
- Circulation—fluid resuscitation and control of bleeding
- Drugs—pharmacotherapy with PPIs, prokinetics, etc.
- Endoscopy (diagnostic and therapeutic) or embolization (therapeutic)

A meta-analysis from Barkun et al. [100] that included forty-one randomized trials showed that all endoscopic therapies decreased rebleeding versus pharmacotherapy alone. Endoscopy is indicated to establish diagnosis and institute therapy for bleeding peptic ulcer [101]. In acutely bleeding ulcers, endoscopy is a part of resuscitation.

**In patients with bleeding peptic ulcer, which are the indications for endoscopic treatment?**

*In patients with bleeding peptic ulcer, we recommend endoscopic treatment to achieve hemostasis and reduce re-bleeding, the need for surgery, and mortality (strong recommendation based on low-quality evidences, 1C).*

*We suggest stratifying patients based on the Blatchford score and adopting a risk-stratified management (weak recommendation based on very low-quality evidences, 2D):*

- *In the very low-risk group, we suggest outpatient endoscopy (weak recommendation based on low-quality evidences, 2C)*
- *In the low-risk group, we recommend early inpatient endoscopy (≤ 24 h of admission) (strong recommendation based on low-quality evidences, 1C).*
- *In the high-risk group, we recommend urgent inpatient endoscopy (≤ 12 h of admission) (strong recommendation based on low-quality evidences, 1C).*

*In patients with spurting ulcer (Forrest 1a), oozing ulcer (Forrest 1b), and ulcer with non-bleeding visible vessel (Forrest 2a), endoscopic hemostasis is recommended (strong recommendation based on low-quality evidences, 1C)*

*In patients with bleeding peptic ulcer, we suggest dual modality for endoscopic hemostasis (weak recommendation based on moderate-quality evidences, 2B)*

*In patients with bleeding peptic ulcer, we suggest considering Doppler probe–guided endoscopic hemostasis if expertise is available (weak recommendation based on very low-quality evidences, 2D).*

Endoscopy not only establishes the diagnosis but also treats the bleeding. WSES advocates patients’ risk determination by using Blatchford score, Forrest classification, and clinical judgment. Three levels of risk stratification are proposed:

- Very low risk—safe for outpatient management, low risk of death
- Low risk—need for admission and early endoscopy
- High risk—need for resuscitation and urgent endoscopy

Risk stratification is based on many risk prediction models and Blatchford score is one of the most validated tools. In an international multicenter prospective study including 3012 patients, Stanley et al. [102] has shown that Blatchford score of 1 or less (very low-risk group) had a sensitivity of 98.6%, specificity of 34.6%, positive predictive value of 96.6%, and a negative predictive value of 56.0% for non-intervention and survival both as the combined endpoint. They also reported that a threshold Blatchford score of 7 or more (high-risk group) was best at predicting endoscopic treatment, with a sensitivity of 80.4%, specificity of 57.4%, positive predictive value of 31.3%, and negative predictive value of 92.4%. Endoscopy reassures a safe and early discharge in low-risk patients and assists therapy in high-risk patients. While the timing of endoscopy is determined by local protocols and resources, the sooner the better, WSES advocates to perform endoscopy at the earliest available opportunity regardless of the risk profile and the only limitation would be resources and expertise. Endoscopy by the “clock” is mere guidance, and if endoscopy could be done earlier, then a clinician should do it. Endoscopy is a part of the resuscitative strategy and blood transfusion should not replace early hemostasis. Dual modality of endoscopic hemostasis is advocated in preference to single modality. Marmo et al. has conducted a meta-analysis [103] including 20 randomized controlled trials and 2472 patients comparing dual therapy versus monotherapy in endoscopic treatment of high-risk bleeding ulcers and concluded that dual endoscopic therapy was superior to epinephrine injection alone in improving outcomes of patients with high-risk bleeding ulcers. In a Cochrane review including 19 randomized studies and 2033 patients, Vergara et al. [104] has shown that additional endoscopic treatment after epinephrine injection reduces further bleeding and the need for surgery in patients with high-risk bleeding peptic ulcer; however, they cannot conclude that a particular form of dual-modality treatment is equal or superior to another. Shi et al. have performed a network meta-analysis on dual therapy choices [105] and shown that the addition of mechanical therapy after epinephrine injection significantly reduced the probability of rebleeding (OR 0.19, 95% CI 0.07–0.52) and surgery (OR 0.10, 95% CI 0.01–0.50). Epinephrine with thermal therapy was shown to reduce the rebleeding rate (OR 0.30, 95% CI 0.10–0.91) but not the need for surgical intervention (OR 0.47, 95% CI 0.16–1.20). Hence, it appears that mechanical therapy along with epinephrine injection is adequate. In patients with adherent clot (Forrest 2b), WSES advocates non-aggressive clot irrigation-flushing attempts rather than mechanical dislodgment. The Asia-Pacific Working Group consensus advocates vigorous target irrigation for at least 5 min and dual-modality hemostasis for patients with adherent clots [106]. We advocate a cautious approach for dislodging the adherent clots. If expertise is available, a vigorous approach could be adopted [107]. The individual endoscopist should be at the liberty to make decisions and we propose individual judgment until further evidence is available to support that clot dislodgment improves outcomes. In the event of bleeding, therapy is strongly advocated. Newer modalities such as over the scope clips (OTSC), hemospray, EUS-guided ultrasound angiography, RFA, Endoclot, endoscopic band ligation, cryotherapy, Ankaferd blood stopper, and endoscopic suturing devices are available. Their role needs to be defined. There are six studies that have investigated the role of over the scope clips either as first-line or as second-line therapy for refractory bleeding [108–113]. Doppler probe–guided lesion assessment is more accurate than endoscopic scoring of predicting rebleeding risk. In a prospective cohort study including 163 patients, Jensen et al. showed spurting (Forrest 1a), visible vessel (Forrest 2a), and adherent clot (Forrest 2b) have a higher Doppler flow compared with oozing (Forrest 1b); Doppler assessment improved risk stratification [114]. It is important to note that rebleeding risk prediction is superior to Forrest classification system, i.e., Forrest 1b has low risk of rebleeding compared with Forrest 2a and Forrest 2b lesions. Doppler probe–guided lesion management is shown to reduce rebleeding and further intervention. In a single blinded randomized controlled study including 148 patients with 125 ulcers, Jensen et al. has shown that Doppler probe–guided endoscopic hemostasis significantly reduced 30-day rates of rebleeding compared with standard, visually guided hemostasis with the number needed to treat of 7 [115].

**In patients with bleeding peptic ulcer, what is the appropriate pharmacological regimen (erythromycin, PPI, terlipressin, others)?**

*In patients with bleeding peptic ulcer, we suggest administering pre-endoscopy erythromycin (weak recommendation based on moderate-quality evidences, 2B).*

*In patients with bleeding peptic ulcer, we suggest starting PPI therapy as soon as possible (weak recommendation based on moderate-quality evidences, 2B),*

*In patients with bleeding peptic ulcer, after successful endoscopic hemostasis, we suggest administration of high-dose PPI as continuous infusion for the first 72 h (weak recommendation based on moderate-quality evidences, 2B).*

*In patients with bleeding peptic ulcer, we recommend PPI for 6–8 weeks following endoscopic treatment. Long-term PPI is not recommended unless the patient has ongoing NSAID use (strong recommendation based on moderate-quality evidences, 1B)*

The role of acid suppression in the treatment of peptic ulcer and its complications is well known [116], but the dosage and the duration of PPI administration for the treatment of bleeding peptic ulcer are still a matter of debate. Multiple studies highlighted that high-dose regimens of PPI [117] reduce rebleeding, surgical intervention, and mortality following endoscopic hemostasis. In a randomized placebo-controlled trial of 767 patients with peptic ulcer bleeding treated with endoscopic therapy because of high-risk stigmata, high-dose intravenous PPIs (80 mg of esomeprazole bolus plus 8 mg/h of continuous infusion for 72 h) significantly reduced rebleeding (5.9% vs. 10.3%, *p* = 0.03) and the need for endoscopic retreatment [118]. Similar results were found by meta-analysis; high-dose intravenous PPIs after endoscopic therapy significantly reduced rebleeding, need for surgery, and mortality compared with placebo/no therapy [119]. On the other hand, a Cochrane review [120] focusing on this topic and including twenty-two RCTs found insufficient evidence to conclude superiority, inferiority, or equivalence of high-dose PPI treatment over lower doses in peptic ulcer bleeding. Another systematic review from the Cochrane Collaboration [121] included six RCTs comprising 2223 patients and showed that PPI treatment initiated before endoscopy for upper gastrointestinal bleeding might reduce the proportion of patients with stigmata of recent bleeding at index endoscopy and significantly reduces the requirement for endoscopic therapy during index endoscopy. However, this study found no evidence that PPI treatment affects clinically important outcomes, namely mortality, rebleeding, or need for surgery. In the light of the above, the administration of high-dose PPI, starting prior to endoscopy and continuing for the first 72 h, seems reasonable and could be suggested, even though further studies are needed to give a strong recommendation. However, the use of proton-pump inhibitors should not replace urgent endoscopy in patients with active bleeding.

A prokinetic drug given before endoscopy helps to empty stomach contents and improves viewing at endoscopy. Only five randomized trials and their pooled analyses have been published: three with the use of erythromycin and two with metoclopramide [122]. Pre-endoscopy erythromycin has been extensively studied and shown to enhance the visualization as well as reduce the need for second endoscopy [123, 124]. However, such practice has not shown to reduce the need for surgical intervention or impact mortality [125].

After initial hemostasis, the risk of rebleeding must be minimized by adjunct therapies. In patients who have PPU complicated by bleeding, there is a 33% risk of rebleeding in 1–2 years. Furthermore, there is a 40–50% rebleeding risk over the subsequent 10 years following the initial episode of bleeding [126]. PPIs are recommended for 6–8 weeks following endoscopic treatment of peptic ulcer bleeding to allow mucosal healing [127]. Once mucosal healing has been achieved, how long PPIs should be continued is still controversial. Randomized prospective trials have demonstrated a benefit to long-term acid-suppression therapy in two settings: chronic NSAID users and *H. pylori*-infected patients [128]. Testing for *H. pylori* is recommended in all patients with BPU. This should be followed by eradication therapy for those who are *H. pylori* positive, with subsequent assessment of the effect of this therapy, and renewed treatment in those in whom eradication fails.

**In patients with recurrent bleeding from peptic ulcer, what is the role of non-operative management?**

*In patients with recurrent bleeding from peptic ulcer, we recommend endoscopy as a first-line treatment (strong recommendation based on low-quality evidences, 1C).*

*In patients with recurrent bleeding, we suggest transcatheter angioembolization as an alternative option where resources are available (weak recommendation based on very low-quality evidences, 2D).*

Emergency endoscopy is the first-line management for rebleeding peptic ulcer [129]. Such endoscopy must be done at the earliest available opportunity. In patients who are hemodynamically stable, angioembolization of the bleeding vessel is an option. However, this should be carefully balanced for its inherent risks of patient transfer, contrast nephropathy, pancreatitis, or cholecystitis risk due to embolization material and risks associated with vascular access.

### Angiography, embolization

**In patients with bleeding peptic ulcer, which are the indications for angiography?**

*In patients with bleeding peptic ulcer, we suggest considering angiography for diagnostic purposes as a second-line investigation after a negative endoscopy (weak recommendation based on low-quality evidences, 2C).*

*No recommendation can be made regarding the role of provocation angiography.*

Angiography may assist both the diagnosis and the treatment of hemorrhage associated with peptic ulcer disease. However, endoscopy remains the first-line investigation of choice for an undifferentiated upper gastrointestinal hemorrhage [130]. Similarly, endoscopy is the first-line diagnostic modality for patients with suspected upper gastrointestinal hemorrhage from ulcer disease [130].

Angiography for diagnostic purposes is a second-line investigation and angiography before endoscopy results in unacceptable rates of negative investigations and is not warranted given the invasive nature of an angiogram. Angiography is useful for the confirmation and localization of the point of hemorrhage and allows treatment by embolization. On occasion, provocation angiography with the use of anticoagulants may be indicated. An inter-specialty consensus should guide this investigation on a case by case basis. Only case reports, case series, and expert opinion are available to guide this decision-making.

**In patients with bleeding peptic ulcer, which are the indications for angioembolization?**

*In hemodinamically stable bleeding peptic ulcer patients, where endoscopic hemostasis fails twice or is not possible/feasible, we suggest angiography with angioembolization where technical skills and equipment are available (weak recommendation based on very low-quality evidences, 2D)*

Endoscopy is the established first-line therapy for the management of hemorrhage associated with peptic ulcer disease. It is appropriate (high-level evidence), to also conduct a second endoscopic examination with therapeutic intent, in cases of recurrent hemorrhage. However, where this also fails, surgery has been traditionally indicated. These operations are reported to be associated with mortality rates as high as 40% [129, 131]. Because of this high postoperative mortality, other strategies have been sought and angioembolization has become increasingly described during the past two decades.

High-risk surgical patients have been suggested and recommended as the ideal candidates for angioembolization [130, 132]. However, no specific data exist investigating or defining the definition of “high risk.” Interdisciplinary consensus (surgery, gastroenterology, intensive care, anesthesia) is required to guide this decision-making. Low-risk surgical patients are likely to benefit from an operative strategy due to the likely reduced mortality in this group. No specific studies exist to validate this claim.

Furthermore, according to the physiology behind wound repair, it is possible that angioembolization could complicate a subsequent surgical intervention because of the reduction in the blood flow of the operative field, but no specific data exists to validate this claim.

**Should embolization be considered for unstable patients with bleeding peptic ulcer?**

*We suggest against a routinely use of angioembolization unstable patients. Angioembolization in unstable patients could be s considered  only in selected cases and in selected facilities (weak recommendation based on very low-quality evidences, 2D).*

There are no specific data to address the relative safety of angioembolization compared with surgery in hemodynamically unstable patients. Variable definitions of hemodynamic stability between studies further complicate meaningful recommendations in this field. Successful reports of angioembolization in patients with hemorrhagic shock are described. A recent retrospective case series describing super-selective angioembolization in 51 patients with active gastrointestinal hemorrhage (with 57% of these upper gastrointestinal in nature), demonstrated the possibility of this approach in patients with physiological shock (defined in this study as a systolic blood pressure of < 90 mmHg) [133].

The appropriateness of angioembolization in hemodynamically unstable patients depends on a number of factors, including the timely availability and skills of the angioembolization service, the quality of the initial and ongoing resuscitation, the quality of the peri-procedural and post-procedural intensive care, and patient variables. Furthermore, the presence of a hybrid OR or strict proximity of OR and the angioembolization facility is mandatory for the angiographic approach to unstable patients. A coordinated, interdisciplinary approach (surgery, interventional radiology, gastroenterology, intensive care, and anesthesia) is likely to benefit these critically ill patients, although there are no specific data to validate this hypothesis.

**In patients with recurrent bleeding peptic ulcer, which are the indications for angioembolization?**

*In patients with rebleeding peptic ulcer, we suggest angioembolization as a feasible option (weak recommendation based on low-quality evidences, 2C).*

For recurrent bleeding (defined as re-bleeding after 2 endoscopic therapeutic attempts), angioembolization and surgical options should be considered. Multiple reports and case series of successful angioembolization of hemorrhage from gastroduodenal ulcer disease are reported [134]. However, no high-level studies comparing the outcomes for angioembolization with surgery exist. One prospective and multiple retrospective cohort studies comparing outcomes between patients undergoing angioembolization with those undergoing surgery for rebleeding after failed endoscopic control are available. These studies were summarized in three meta-analysis [135–137]. Kyaw et al. summarized 6 retrospective cohort studies: surgery was found to significantly reduce the likelihood of further (post-intervention) hemorrhage, and was associated with a trend towards a reduced need for further intervention. However, surgery was also associated with a trend to increased mortality. Beggs et al. included 9 cohort studies (8 retrospective and 1 prospective), and similarly concluded that surgery was associated with a significantly lower risk of rebleeding, and only a marginal trend towards increased mortality. Subsequent to these first two meta-analyses, a case-control study comparing angioembolization with surgery [138] reported a trend to higher rebleeding rates following angioembolization, and a trend towards higher mortality after surgery was seen. A significantly lower rate of post-procedural complications was reported in the angioembolization cohort. The latest meta-analysis [137] found similar results, but interestingly found a slight drift toward a lower mortality for the angioembolization group.

**In patients with bleeding peptic ulcer who underwent angioembolization, which are the most appropriate embolization techniques and materials?**

*Varied techniques and materials exist for the use in the embolization of bleeding duodenal ulcer disease. A tailored approach, guided by the multidisciplinary team, incorporating patient, pathology, and environmental factors is suggested (weak recommendation based on low-quality evidences, 2C).*

Successful embolization of gastric and duodenal arteries is complicated by the rich collateral blood supply. Several technical points are raised in various case reports, series, and review articles in this field. There are no high-level articles to guide these technical considerations. Pre-procedural endoscopic localization of the point of hemorrhage could assist guidance of the selective and super-selective angiography and the angiogram can be further guided by the placement of an endoscopic clip at the ulcer if this has been identified. Diagnostic angiography usually commences with a selective coeliac axis and superior mesenteric artery catheterization and angiogram. Where no extravasation is seen, a super-selective approach normally follows. Imaging from both aspects of the bleeding point is ideally obtained (both sides need to be approached).

**In patients with bleeding peptic ulcer and non-evident bleeding during angiography, is there a role for prophylactic embolization?**

*No recommendation can be made on the role of prophylactic embolization.*

Prophylactic embolization may be considered in two situations

- Empirically, at the time of a negative angiogram: Several authors have suggested a role for blind embolization for upper gastrointestinal hemorrhage, noting that these patients had similar outcomes to patients who underwent embolization after the demonstration of a point of hemorrhage [134, 139, 140]. A variation on this uses the endoscopic information to guide the area for embolization [141, 142]. However, these approaches are based on retrospective cohorts. There are insufficient high-level data to draw firm conclusions.
- As a planned intervention, in association with endoscopic control: The addition of prophylactic embolization in addition to endoscopic hemostasis has been investigated by several authors, including most recently with two randomized controlled trials [143, 144]. Laursen et al. demonstrated a trend toward improved outcomes in patients who underwent additional prophylactic embolization. However, the second RCT by Lau et al. failed to confirm this observation. This approach was also supported by a retrospective series by Mille et al. [145].

At present, the evidences available in the literature appear to be insufficient to routinely recommend this approach.

### Surgery

**In patients with bleeding peptic ulcer, which are the indications for surgical treatment and which is the appropriate timing for surgery?**

*In patients with bleeding peptic ulcer, we suggest surgical hemostasis (or angiographic embolization if immediately available and with appropriate skills) after failure of repeated endoscopy. In patients with hypotension and/or hemodynamic instability and/or ulcer larger than 2 cm at first endoscopy, we suggest surgical intervention without repeated endoscopy (strong recommendation based on very low-quality evidences, 1D).*

A renowned RCT conducted in 1999 [129] compared endoscopic retreatment with surgery for peptic ulcer rebleeding after initial endoscopy. Over a 40-month period, 92 patients with recurrent bleeding were enrolled: 48 patients were randomly assigned to undergo immediate endoscopic retreatment and 44 were assigned to undergo surgery. Of the 48 patients who were assigned to endoscopic retreatment, 35 had long-term control of bleeding. Thirteen underwent salvage surgery, 11 because retreatment failed, and 2 because of perforations resulting from thermocoagulation. Five patients in the endoscopy group died within 30 days, as compared with eight patients in the surgery group (*p* = 0.37). Seven patients in the endoscopy group had complications, as compared with 16 in the surgery group (*p* = 0.03). Duration of hospitalization, need for ICU admission, ICU length of stay, and the number of blood transfusions were similar in the two groups. In multivariate analysis, hypotension at randomization (*p* = 0.01) and an ulcer size of at least 2 cm (*p* = 0.03) were independent factors predictive of the failure of endoscopic retreatment. According to these data, repeated endoscopy is indicated for stable patients with ulcers smaller than 2 cm in diameter, while for patients with a larger ulcer and heavier bleeding, surgery may be taken into account as a first-line therapy.

No evidence is available regarding the impact on clinical outcome of time before surgery for bleeding peptic ulcer. We suggest immediate surgery for unstable patients with bleeding peptic ulcer refractory to endoscopy/angioembolization.

**In patients with bleeding peptic ulcer, what is the most appropriate surgical approach (open vs laparoscopy) and what are the most appropriate surgical procedures?**

*In patients with refractory bleeding peptic ulcer, we suggest surgical intervention with open surgery (weak recommendation based on very low-quality evidences, 2D).*

*In patients operated for bleeding peptic ulcer, we suggest intra-operative endoscopy to facilitate the localization of the bleeding site (weak recommendation based on very low-quality evidences, 2D).*

*We suggest choosing the surgical procedure according to the location and extension of the ulcer and the characteristics of the bleeding vessel (weak recommendation based on low-quality evidences, 2C)*

*An immediate or delayed biopsy is recommended (weak recommendation based on low-quality evidences, 2C)*

A refractory bleeding peptic ulcer is defined as an ulcer still bleeding after repeated endoscopy/angioembolization. Open surgery is recommended when endoscopic treatments have failed and there is evidence of ongoing bleeding, plus or minus hemodynamic instability. The choice of the appropriate surgical procedure for bleeding peptic ulcer should be made on the basis of the location and extension of the ulcer and the characteristics of the bleeding vessel. Surgical approach involves ulcer oversew or resection. Bleeding gastric ulcers should be resected or at least biopsied for the possibility of neoplasms. Conversely, most duodenal ulcers requiring surgery for persistent bleeding are usually large and posterior lesions, and the bleeding is often from the gastro-duodenal artery. A recent prospective cohort study conducted in Denmark [146] compared the outcomes of duodenal and gastric bleeding peptic ulcers and found a significantly higher 90-day mortality and reoperation rate for the duodenal location, confirming the greater complexity of surgical management of this ulcer. Via duodenotomy, the bleeding vessel can be seen on the floor of the ulcer and can be rapidly oversewn. It is critical to perform triple-loop suturing of bleeding of the GDA due to the collateral blood supply to the transverse pancreatic arteries. The surgeon may not know preoperatively where the bleeding originates and intraoperative endoscopic guidance may be helpful. For patients with intractable ulcer bleeding, Schroeder et al. [147] from the analysis of a large database (ACS-NSQIP) have found that the surgical procedure of vagotomy/drainage is associated with significantly lower mortality than simply simple local ulcer oversew. They further suggest that vagotomy/drainage is preferred to local procedures alone for the surgical management of patients with bleeding peptic ulcer disease requiring emergency operation for intractable bleeding ulcers.

**In patients with bleeding peptic ulcer, what is the role of damage control surgery?**

*We suggest considering damage control surgery for patients with hemorrhagic shock and signs of severe physiological derangement, in order to quickly resolve the bleeding and allow a prompt ICU admission (weak recommendation based on very low-quality evidences, 2D).*

Indications for damage control surgery in bleeding peptic ulcer are similar to those for perforated peptic ulcer and are reported in the WSES guidelines on Open Abdomen management in non-trauma patients [67].

### Antimicrobial therapy

**In patients with bleeding peptic ulcer, which are the indications for antimicrobial therapy and for** ***Helicobacter pylori*** **testing?**

*In patients with bleeding peptic ulcer, empirical antimicrobial therapy is not recommended (strong recommendation based on low-quality evidences, 1C)*

*We recommend performing Helicobacter pylori testing in all patients with bleeding peptic ulcer (strong recommendation based on low-quality evidences, 1C).*

Bleeding peptic ulcer accounts for 75% of patients admitted to ED for peptic ulcer disease [148] and has different etiologies (ulcerogenic medications such as acetylsalicylic acid and NSAIDs, *H. pylori* infection, etc). *H. pylori* infection has a variable prevalence of 20–50% among patients with bleeding peptic ulcer in various countries, but its eradication is associated with a significant reduction in ulcer recurrence rate and rebleeding [66, 149–151]. In a systematic review, Gisbert et al. showed a 26% rebleeding rate among patients with *H. pylori* infection–associated bleeding ulcers who did not receive eradication therapy [150]. Conflicting results are reported about appropriate timing to start eradication therapy. Empirical eradication therapy immediately after re-feeding has been suggested as the most cost-effective strategy [151], but its real effectiveness can vary by regional prevalence of the bacteria. Therefore, confirming the result of *H. pylori* test and initiating eradication therapy in *H. pylori*-positive patients prior to discharge would appear to be a more appropriate strategy than to apply empirical therapy to all patients with BPU [66, 152].

For this reason, all patients having BPU should undergo *H. Pylori* testing. Different tests are available to confirm *H. pylori* infection. The urea breath test (UBT) and stool antigen testing are acceptable non-invasive tests with a sensitivity of 88–95% for UBT and 94% for stool antigen testing, respectively. Specificity is 95–100% for UBT and 92% for stool antigen testing, respectively [151]. In cases of bleeding peptic ulcer, *H. pylori* testing on endoscopic tissue biopsy may be available [151].

**In patients with bleeding peptic ulcer and positive tests for HP infection, which are the therapeutic options?**

*In H. pylori-positive BPU patients, eradication therapy is recommended to avoid recurrent bleeding (strong recommendation based on low-quality evidences, 1C)*

*In patients with HP positive tests, standard triple therapy (amoxicillin, clarithromycin, and PPI) regimen is recommended as first-line therapy if low clarithromycin resistance is present (strong recommendation based on moderate-quality evidences, 1B)*

*10 days of sequential therapy with four drugs (amoxicillin, clarythromicin, metronidazole, and PPI) is recommended in selected cases, if compliance to the scheduled regimen can be maintained, and if clarithromycin high resistance is detected (strong recommendation based on low-quality evidences, 1C).*

*In patients with HP positive tests, a 10-day levofloxacin-amoxicillin triple therapy is recommended as second-line therapy if first-line therapy failed (strong recommendation based on moderate-quality evidences, 1B).*

*We recommend to start standard triple therapy (STT) after 72–96 h of intravenous administration of PPI and to administer it for 14 days (strong recommendation based on low-quality evidences, 1C)*

The worldwide prevalence of *H. pylori* infections is approximately 50%, with the highest being in developing countries [153]. Standard treatments for *H. pylori* infections have been endorsed by Western scientific societies, and by regulatory authorities relying on clarithromycin, metronidazole, or amoxicillin in conjunction with PPI [154].

As the response to eradication therapy is significantly related to the prevalence of primary resistance in the population, the choice of treatment regimen should be based on the knowledge of the underlying prevalence of resistant strains in the community [151–154].

Several international guidelines [151, 152] and available meta-analysis [153, 154] recommend that standard triple therapy (amoxicillin, clarithromycin, and PPI) regimen should be used as first-line therapy if low clarithromycin resistance is present. The suggested doses are:

- PPI standard dose twice a day;
- Clarithromycin 500 mg twice a day;
- Amoxicillin 1000 mg twice a day, or
- Metronidazole 500 mg twice a day.

Sequential therapy with four drugs (amoxicillin, clarithromycin, metronidazole, and PPI) should be considered in selected cases, if compliance to the scheduled regimen can be maintained, and if clarithromycin high resistance is detected. It is defined as the use of one PPI and amoxicillin for the first 5 days followed by PPI plus clarithromycin and metronidazole for the next 5 days [155]. Recommended doses are as follows:

- PPI standard dose twice a day;
- Amoxicilllin 1000 mg twice a day;
- Clarithromycin 500 mg twice a day;
- Metronidazole 500 mg twice a day.

If any of these regimens failed, a second-line therapy is represented by a 10-day levofloxacin-amoxicillin triple therapy. The suggested doses are:

- PPI standard dose twice a day;
- Levofloxacin 500 mg once a day or 250 twice a day;
- Amoxicillin 1000 mg twice a day.

## Conclusions

Peptic ulcer disease is still common among the world population and its incidence pattern is evolving in relation to the rise of new risk factors, i.e., the increasing incidence of the *Helicobacter pylori* infection, the extensive use of NSAIDs and the increase in alcohol and smoking abuse. Despite the tremendous improvement in preventive therapies, the rate of complication of this disease is still high and is burdened by high morbidity and mortality. Prompt recognition and treatment of the complications lead invariably to a better outcome, especially in elderly and frail patients. For this reason, these guidelines present evidence-based international consensus statements on the management of complicated peptic ulcer from a collaboration of a panel of experts and are intended to improve the knowledge and the awareness of physicians around the world on this specific topic. We divided our work into two main topics, bleeding and perforated peptic ulcer, and structured it into six main topics that cover the entire management process of patients with complicated peptic ulcer, from diagnosis at ED arrival to post-discharge antimicrobial therapy, to provide an up-to-date and easy-to-use tool that can help physicians and surgeons during the decision-making process.

## Data Availability

The authors are responsible for the data described in the manuscript and assure full availability of the study material upon request to the corresponding author.

## Abbreviations

ACS-NSQIP: American college of surgeon National Surgical Quality Improvement Program
APACHE score: Acute Physiology and Chronic Health Evaluation score
ASA score: American Society of Anesthesiologists score
ASA: Acetylsalicylic acid
ATLS: Advanced trauma life support
AXR: Abdominal X-ray
BPU: Bleeding peptic ulcer
COOL trial: Closed or Open after Laparotomy trial
CPU: Complicated peptic ulcer
CT: Computed tomography
ED: emergency department
EUS: Endoscopic ultrasound
GBS: Glasgow-Blatchford score
GI: Gastrointestinal
GRADE: Grading of Recommendations Assessment, Development and Evaluation
Hb: Hemoglobin
IAI: intra-abdominal infection
ICU: Intensive care unit
INR: International normalized ratio
MAP: Mean arterial pressure
MDRO: Multi-drug resistant organism
NGT: Nasogastric tube
NOM: Nonoperative management
NSAIDs: Non-steroidal anti-inflammatory drugs
OA: Open abdomen
OR: Operating room
OTSC: Over the scope clip
PPI: Proton-pomp inhibitor
PPU: Perforated peptic ulcer
PULP score: Peptic ulcer perforation score
qSOFA: Quick sequential organ failure assessment
RCT: randomized controlled trial
RFA: Radiofrequency ablation
SOFA: Sequential organ failure assessment
SSI: Surgical site infection
UBT: Urea breath test
WSES: World Society of Emergency Surgery
